# Integrating human stem cell expansion and neuronal differentiation in bioreactors

**DOI:** 10.1186/1472-6750-9-82

**Published:** 2009-09-22

**Authors:** Margarida Serra, Catarina Brito, Eunice M Costa, Marcos FQ Sousa, Paula M Alves

**Affiliations:** 1 Instituto de Tecnologia Química e Biológica, Universidade Nova de Lisboa, Av. da República, 2780-157 Oeiras, Portugal; 2 IBET, Apartado 12, 2781-901 Oeiras, Portugal

## Abstract

**Background:**

Human stem cells are cellular resources with outstanding potential for cell therapy. However, for the fulfillment of this application, major challenges remain to be met. Of paramount importance is the development of robust systems for *in vitro *stem cell expansion and differentiation. In this work, we successfully developed an efficient scalable bioprocess for the fast production of human neurons.

**Results:**

The expansion of undifferentiated human embryonal carcinoma stem cells (NTera2/cl.D1 cell line) as 3D-aggregates was firstly optimized in spinner vessel. The media exchange operation mode with an inoculum concentration of 4 × 10^5 ^cell/mL was the most efficient strategy tested, with a 4.6-fold increase in cell concentration achieved in 5 days. These results were validated in a bioreactor where similar profile and metabolic performance were obtained. Furthermore, characterization of the expanded population by immunofluorescence microscopy and flow cytometry showed that NT2 cells maintained their stem cell characteristics along the bioreactor culture time.

Finally, the neuronal differentiation step was integrated in the bioreactor process, by addition of retinoic acid when cells were in the middle of the exponential phase. Neurosphere composition was monitored and neuronal differentiation efficiency evaluated along the culture time. The results show that, for bioreactor cultures, we were able to increase significantly the neuronal differentiation efficiency by 10-fold while reducing drastically, by 30%, the time required for the differentiation process.

**Conclusion:**

The culture systems developed herein are robust and represent one-step-forward towards the development of integrated bioprocesses, bridging stem cell expansion and differentiation in fully controlled bioreactors.

## Background

Many neurodegenerative disorders, such as Parkinson's disease, are caused by the impairment or death of neurons in the central nervous system [1]. In the future, it is hoped that large numbers of stem cell-derived neurons will be produced in culture with the purpose of being used in clinical applications [2]. Hampering the faster implementation of the ambitious stem cell therapy technology, there is still the need of efficient, robust and scalable bioprocesses for cell expansion and/or differentiation *in vitro*.

During the last five years, substantial progress has been made towards this goal [3,4]. Stirred suspension systems have been pioneered, by others and ourselves, as a promising *in vitro *system for stem cell expansion [5,6], embryoid body cultivation [7,8] and stem cell differentiation into specific cell types [9]. These systems offer attractive advantages of scalability and relative simplicity; stirring provides a more homogenous culture environment and allows the measurement and control of extrinsic factors such as nutrient and cytokine concentration, pH and dissolved oxygen (pO_2_) [10].

Aiming to improve the yields of specific stem cell stages, several culture parameters have been optimized, including the agitation rate, cell inoculum concentration and medium composition [3,4,11], and different culturing approaches have been developed such as the use of microcarrier supports [5] and cell encapsulation [11]. Perfusion and frequent feeding operation modes have been shown to increase the expansion of mesenchymal stem cells [11], embryonic stem cells [12,13] and mammary epithelial stem cells [14], without compromising their stem cell performance.

Computer-controlled bioreactors are particular advantageous for process development by allowing the online monitoring and control of specific culture parameters (temperature, pH and pO_2_), ensuring a fully controlled environment for stem cell cultivation. Oxygen-controlled bioreactors have been used for culture of mouse and human ESC-derived cardiomyocytes [7,15]. Gilbertson *et al *[16] were the first group to use controlled conditions for neural precursor cell culture as aggregates; the authors report the successful expansion of mouse neural stem cells in 500 mL bioreactors (temperature, pH and pO_2 _control) while retaining the cell multilineage potential [16]. More recently, this system was applied to the culture of human neural precursor cells [17]. The expansion of various human stem cell types in bioreactors under defined and controlled conditions remains to be addressed. Future challenges also include the combination of expansion and directed differentiation steps in an integrated bioprocess that will ultimately result in scale-up of well differentiated cells to clinically relevant numbers.

Within this context, the present work focused the development of a reproducible scalable system for the production of human neurons derived from expanded and differentiated stem cells. The human embryonal carcinoma cell line NTera-2/cl.D1 (NT2) was the cellular system used because it is a valuable model for both undifferentiated human embryonic stem cells (hESCs) [18] and human neuronal differentiation *in vitro *[19]. In addition, the neurons derived from this cell line have been successfully used in transplantation studies in several mouse models and in human stroke patients [20], providing also promising material for cell therapy investigations in central nervous system.

Herein, undifferentiated NT2 cells were cultivated as 3D-aggregates in controlled stirred suspension conditions. In order to improve the yields of stem cells, two parameters were studied: (i) the inoculum concentration, as it has been shown to be critical in enhancing cell aggregation and culture profile [6], and (ii) the culture operation mode, since it has been demonstrated that the feeding strategy affects cell metabolism and consequently could improve cell culture performance [11,15,21]. At the end, the expansion of undifferentiated NT2 cells, followed by directed neuronal differentiation were integrated in stirred bioreactors with temperature, pH and pO_2 _control, in an effort to develop a promising model system for the production of human stem cell derivatives.

## Results

**Figure 1:**
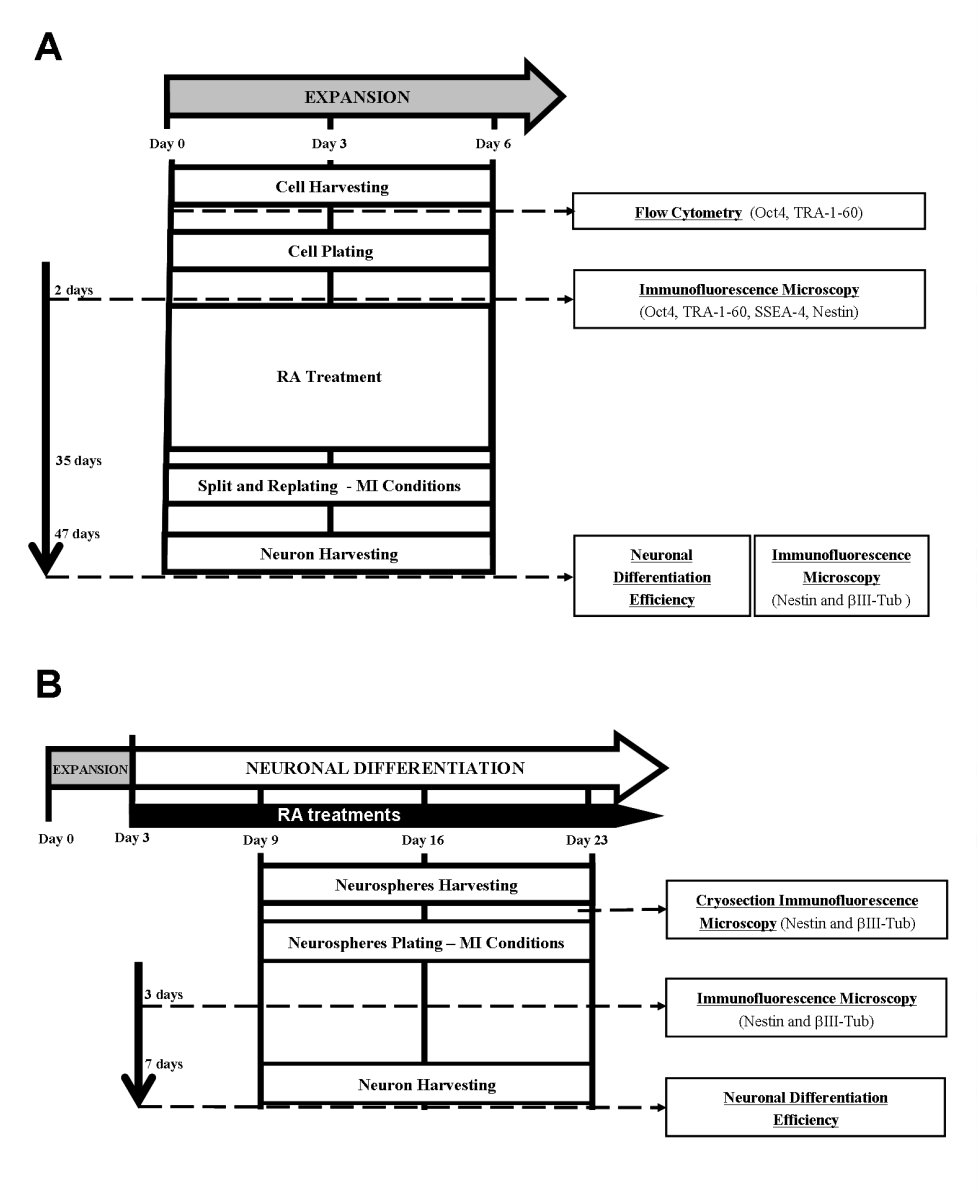
**Experimental outline for NT2 cell sampling and characterization during expansion (A) and differentiation (B) in fully controlled bioreactors**. (A) In expansion runs, cells were harvested from days 0 (inoculum), 3 and 6 and immediately characterized by flow cytometry. Harvested cells were plated on glass coverslips and processed for immunofluorescence microscopy analysis after 2 days or plated in tissue culture flasks for induction of neuronal differentiation. For this, cultures were treated with retinoic acid (RA) for 5 weeks, splitted and further cultured in mitosis inhibitory (MI) conditions. After 12 days in MI, the neurons were harvested, identified by immunofluorescence microscopy using neuronal markers and neuronal differentiation efficiencies were calculated. (B) In differentiation runs, the addition of RA was initiated at day 3 of bioreactor culture and prolonged for 3 weeks. Neurospheres were harvested at day 9, 16 and 23. The latest were analyzed by cryosection immunofluorescence microscopy. All neurosphere harvested were plated in static culture flasks and cultured in MI conditions. After 3 days, cultures were characterized by immunofluorescence microscopy and after 7 days and neuronal differentiation efficiencies were calculated.

With the goal of developing a robust and scalable system for NT2 neuronal differentiation, both expansion and differentiation steps were integrated in a fully controlled bioreactor process. Firstly, different strategies for expansion of undifferentiated NT2 cells as 3-D aggregates were screened in stirred spinner vessels; two parameters were studied (i) the inoculum concentration and (ii) the culture operation mode, i.e., medium replenishing strategies. Having the expansion of pluripotent NT2 cells optimized and well characterized, the neuronal differentiation strategy previously developed by our group [9], was integrated and the overall bioprocess combined in the bioreactor. Figure 1 summarizes the experimental outline used for expansion and differentiation processes.

### Effect of inoculum concentration in NT2 expansion

Three different cell inoculum concentrations were tested in batch culture mode, using 125 mL spinners: 0.4, 1 and 4 × 10^5 ^cell/mL (SP-0.4B, SP-1B and SP-4B, respectively).

**Figure 2:**
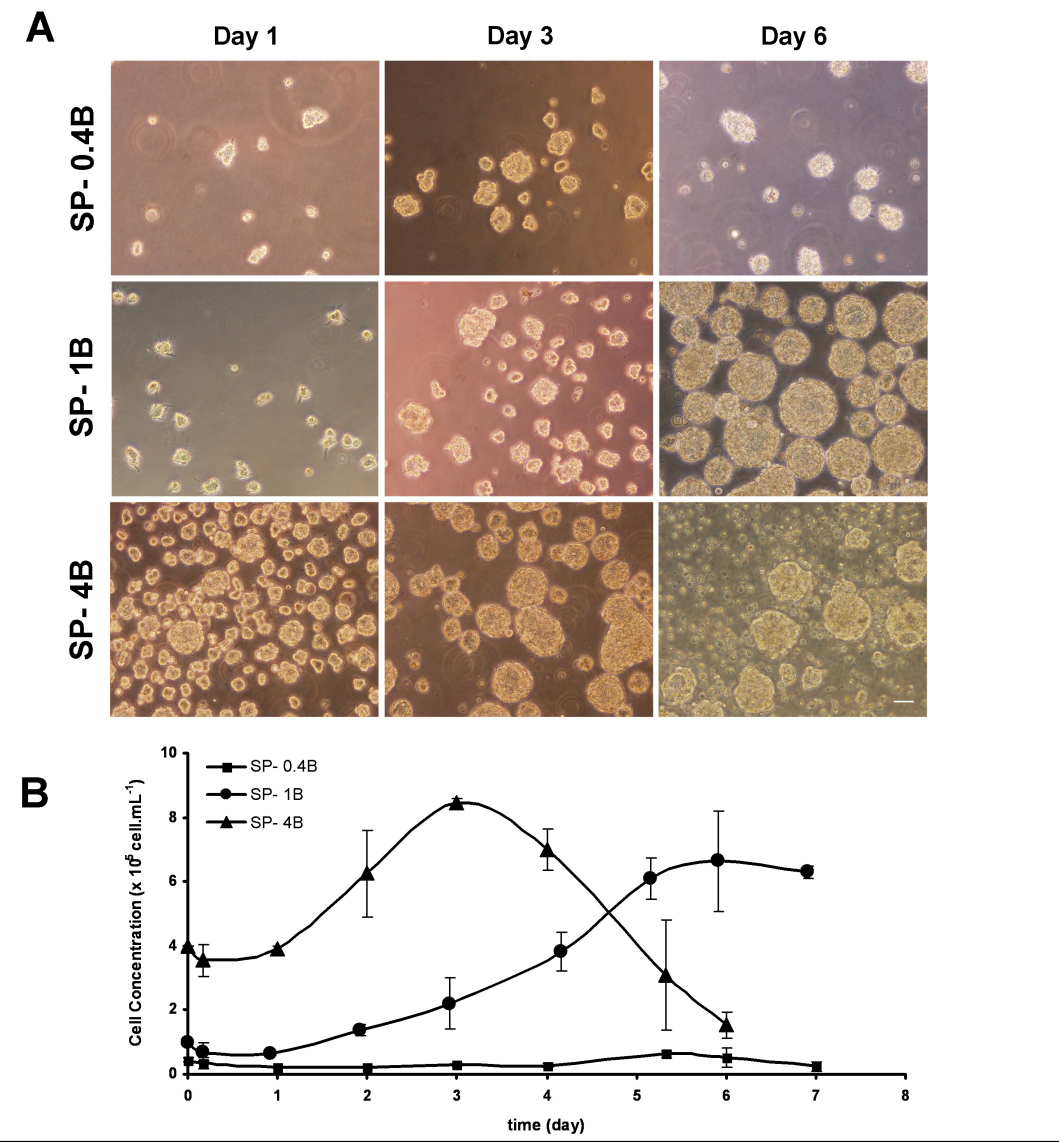
**Effect of inoculum concentration in NT2 cell expansion as 3D-aggregates**. Cells were cultured in spinner vessels with inoculum concentrations of 0.4 (SP-0.4B, squares), 1 (SP-1B, circles) and 4 (SP-4B, triangles) ×10^5 ^cell/mL. Phase contrast photomicrographs of cultures samples visualized by day 1, day 3 and day 6 of cultivation. Scale bar: 100 μm (**A**). Growth curves expressed in terms of cell concentration; error bars denote standard deviation of average from 2 independent experiments (**B**).

During the first 24 h of SP-1B and SP-4B cultures, cells assembled into small 3D-aggregates (Figure 2A) ranging from 40 to 65 μm. After this period, cells started to divide and aggregate size increased up to 150 μm. The growth curve and the calculated apparent growth rates are shown in Figure 2B and Table 1, respectively. SP-1B exhibited a high apparent growth rate (0.51 ± 0.01 day^-1^) and the highest FI in cell concentration (7.14 ± 0.86). Nevertheless, maximum cell density 6.64 (± 1.57) × 10^5 ^cell/mL was only reached 6 days after inoculation, whereas in SP-4B, a maximum of 8.48 (± 0.11) × 10^5 ^cell/mL was achieved at day 3. From day 4 onwards of SP-4B culture, cells started to detach from the aggregates (Figure 2A), resulting in cell death (data not shown). Similar behavior was observed for SP-1B culture upon day 7 of cultivation.

**Table 1:** Growth kinetics of NT2 cell expansion as 3D-aggregates using different culture strategies.

**Strategy**	**μ (day^-1^)**	**FI**	**X_max _(×10^5 ^cell/mL)**
SP-0.4B	n. a.	n. a.	0.63 ± 0.11 *
SP-1B	0.51 ± 0.01	7.14 ± 0.86 *	6.64 ± 1.57
SP-4B	0.39 ± 0.02	2.12 ± 0.03	8.48 ± 0.11
SP-4FB	0.52 ± 0.06	4.30 ± 0.33 *	17.19 ± 1.30 *
SP-4ME	0.41 ± 0.06	4.56 ± 0.04 *	18.25 ± 0.18 *
BR-4ME	0.37 ± 0.03	4.10 ± 0.41	16.25 ± 0.16

Apparent growth rate (μ), fold increase (FI) and maximum cell concentration values (X_max_) of NT2 cells cultured in spinner vessel (SP) or in bioreactor (BR); with inoculum densities of 0.4 × 10^5 ^(SP-0.4) 1 × 10^5 ^(SP-1) or 4 × 10^5 ^cell/mL (SP-4, BR-4); in batch (B), fed-batch (FB) and media-exchange (ME) culture operation mode. Results are expressed as mean ± SEM from n = 2 independent experiments. n. a. - not applicable. *Indicates significant statistical difference (p-value < 0.05) from the SP-4B mean values of μ, FI and Xmax by the one-way ANOVA analysis with a Scheffé post-hoc multiple comparison test.

Concerning the SP-0.4 culture, cell aggregates were rare and small throughout cultivation time (Figure 2A). In fact, no effective cell growth was observed (Figure 2B) and cell viability was low (data not shown).

Aiming to develop an efficient bioprocess for the fast production of human neurons, cell number and culture time were the parameters preferentially used to select the best strategy. For SP-4B, the time needed to achieve X_max _was 2 times lower than for SP-1B, reaching similar X_max _values (Table 1). Based on these results, SP-4B was chosen to be further optimized and integrated with the neuronal differentiation step.

### Impact of operation mode in NT2 cell expansion

**Figure 3:**
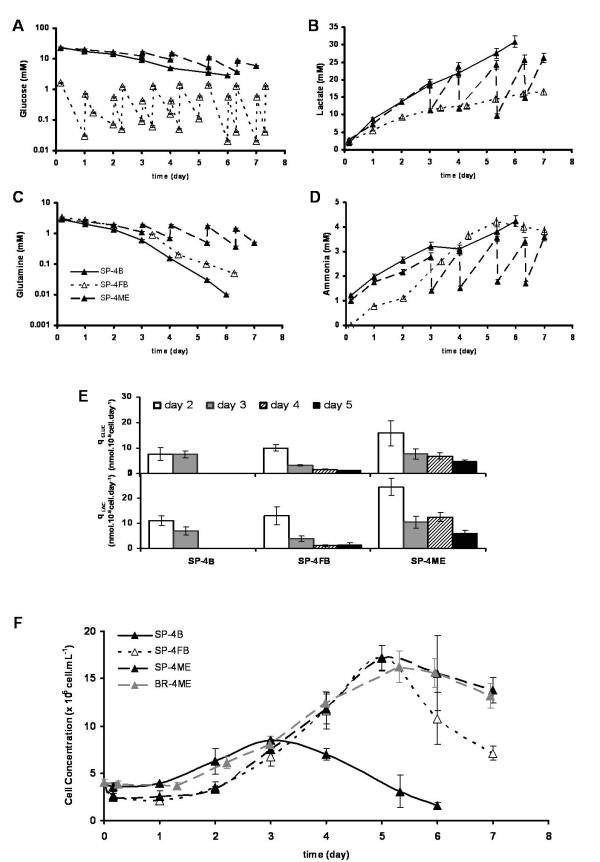
**Effect of culture operation mode on NT2 cell expansion as 3D-aggregates**. Cells were cultured in spinner vessels (SP) or in bioreactors (BR), with inoculum concentration of 4 × 10^5 ^cell/mL, using different operation modes: batch (SP-4B, black line and triangles), fed-batch (SP-4FB, dashed line and white triangles) and media exchange (SP-4ME, dashed line and black triangles, and BR-4ME, grey line and triangles). Concentrations of glucose **(A)**, lactate **(B)**, glutamine **(C) **and ammonia **(D) **presented in media during culture time. Specific rates of glucose consumption and lactate production shown over the course of exponential growth phase **(E) **(day 2- white bars, day 3- grey bars, day 4-striped bars, day 5- black bars). Growth curves expressed in terms of cell concentration; error bars denote standard deviation of average from 2 independent experiments **(F)**.

In all batch cultures there was a rapid decrease in cell density after the culture reached its maximum concentration value (Figure 2A). Although no complete depletion of neither glucose nor glutamine was observed (Figure 3A, C), this profile could be correlated to the exhaustion of other essential nutrients and/or the progressive accumulation of toxic metabolic waste products such as lactate and ammonia (Figure 3B, D). In SP-4B, by the 4^th ^day of cultivation, the lactate and ammonia concentrations were already 21.9 mM and 3.1 mM, respectively (Figure 3B, D). In SP-1B, these values were also high at day 7 of culture (27.2 mM and 4.2 mM for lactate and ammonia concentration, respectively).

Aiming at prolonging the exponential growth phase and improve the cell expansion, two additional operation modes were tested. The first strategy consisted of a glucose fed-batch operation mode (SP-4FB). In this strategy, culture was initiated at low concentration of glucose (1.4 mM) and the feeding was performed twice a day assuring the maintenance of low levels of glucose throughout cultivation time (see Methods section). The second strategy (SP-4ME) was designed to simulate a perfusion system, in which cells are kept in culture and the media is renovated regularly. This was achieved by performing a daily partial media exchange (50%) from the 3^rd ^cultivation day onwards, as this time point corresponded to the growth peak in the batch culture (Figure 2B, SP-4B).

For SP-4ME and SP-4FB cultures, the exponential growth phase was extended until day 5 (Figure 3F), with a significant increase in X_max_, when compared to SP-4B (Table 1). These differences are also reflected in cell metabolism, as shown by the nutrient consumption and metabolite production profiles (Figure 3E). The SP-4FB culture presented the lowest specific rates of glucose consumption and lactate production. The lower accumulation of lactate (16.5 mM at day 6, Figure 3B) in SP-4FB contributed to the high apparent growth rate of this strategy (0.52 ± 0.06 day^-1^, Table 1). Nevertheless, there was still a steeply decrease in cell concentration after day 6 (Figure 3F) that may result from the accumulation of other toxic metabolites, such as ammonia, which reached values as high as in SP-4B (4.0 mM and 4.2 mM for SP-4FB and SP-4B cultures, respectively, at day 6 of cultivation, Figure 3D).

Cell viability was calculated in term of cell lysis, translated by the specific release rates of the intracellular enzyme LDH (q_LDH_). For SP4-ME, the q_LDH _achieved were lower (fold increase of 9.1) than those obtained for SP-4B and SP-4FB (fold increase of 20.5 and 19.4, respectively) throughout 6 days of cultivation, indicating that a lower percentage of cell lysis occurred in the SP-4ME culture. Despite no complete depletion of either glucose or glutamine was observed in the strategies tested, cells in SP4-ME were not continuously subjected to the accumulation of toxic metabolites, which probably had a positive effect on cell viability (Figure 3A-D).

### Expansion and characterization of undifferentiated NT2 cells in a bioreactor

From the results shown above, SP-4ME was the most promising culture strategy for expansion of undifferentiated stem cell. The next step was the implementation of this strategy in a fully controlled 125 mL bioreactor, BR-4ME.

**Figure 4:**
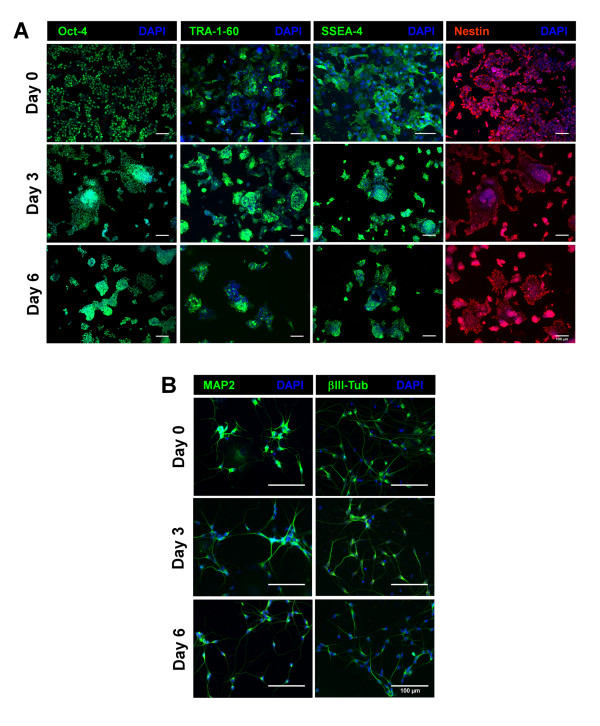
**Characterization of NT2 cells expanded as 3D-aggregates**. Immunofluorescence images of cells from the inoculum (day 0) and collected from the bioreactor culture (day 3 and day 6). Immunolabeling of Oct-4, TRA-1-60, SSEA-4 (green) and nestin (red). Nuclei are labeled with DAPI (blue) (**A**). Immunofluorescence images of differentiated cultures derived from the inoculum (day 0) and from the bioreactor culture (day 3 and day 6). Neurons labeled with βIII-Tub and MAP2 (green) (**B**). Nuclei were stained with DAPI (blue). Scale bars: 100 μm.

The growth curve obtained for the bioreactor run BR-4ME was comparable to the one obtained for the medium exchange operation mode in spinner SP-4ME; similar apparent growth rates and maximum concentrations were obtained (Figure 3F, Table 1). NT2 cells expanded in the bioreactor for 6 days were characterized in terms of pluripotency, undifferentiated phenotype and differentiation potential. The expression of stem cell markers (Oct-4, TRA-1-60, SSEA-4) and nestin, an intermediate filament protein associated with undifferentiated phenotype of NT2 cells [22], was detected during exponential growth phase (day 3) and at day 6 (Figure 4A). This labeling pattern was similar to the cell inoculum (day 0).

Moreover, in addition to the expression of stem markers analysis, the expanded cells ability to differentiate into neurons was also confirmed. For that purpose, cells were collected at 3 time points (day 0, 3 and 6) and induced to differentiate into neurons using the standard static differentiation protocol [23]. After treatment with RA and further cultivation in MI medium, the neuronal differentiation efficiency (defined as the ratio between the number of neurons obtained and the number of cells harvested from the bioreactor, see Methods section) was similar for all culture samples, presenting values in the range typically obtained for the static differentiation protocol (3.3 ± 0.2%) [9]. The differentiated neurons were identified by βIII-Tub and MAP2 positive staining (Figure 4B).

Overall, these results showed that NT2 cells maintained their pluripotency, undifferentiated phenotype, and differentiation potential along expansion in the bioreactor.

### Integrating expansion and neuronal differentiation of NT2 cells in the bioreactor

Once the expansion of pluripotent NT2 cells was adapted and characterized in the bioreactor system, we further integrated the neuronal differentiation step according to Serra et al [9]. Neuronal differentiation was induced by RA addition when cells achieved the middle of the exponential growth phase at day 3 (Figure 3C). Flow cytometry analysis of cell populations showed that the levels of Oct-4 (94.8% positive cells) and Tra-1-60 (88.7% positive cells) obtained for the inoculum were kept at day 3 of the bioreactor culture (97.2% and 94.6% Oct-4 and Tra-1-60 positive cells, respectively), confirming that the stem cell population was maintained at this time point.

**Figure 5:**
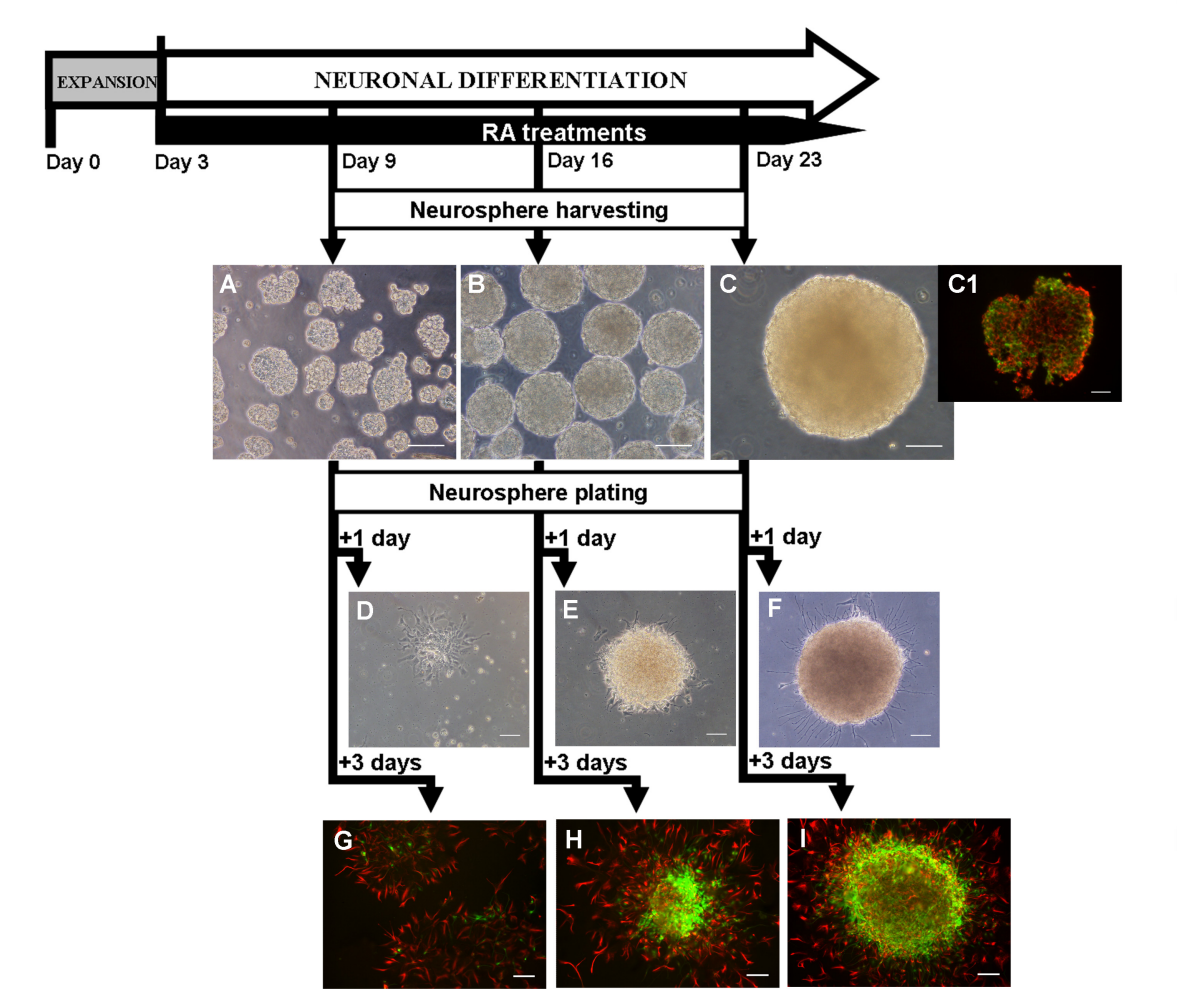
**Neuronal differentiation of NT2 cells in a fully controlled bioreactor**. Neuronal differentiation was induced by addition of retinoic acid (RA) from day 3 onwards (RA treatments). Phase contrast photomicrographs of neurospheres harvested at day 9 (**A**), day 16 (**B**) and day 23 (**C**) of the bioreactor culture. By day 23, neurosphere composition was analyzed by cryosection immunofluorescence microscopy - double labeling of nestin (red) and βIII-Tub (green) (**C1**). Harvested neurospheres were further cultured in mitotic inhibitory (MI) conditions, on poly-D-lysine and Matrigel-coated surfaces. Cultures were visualized by phase contrast microscopy 1 day after plating (**D**,**E**,**F**) and characterized by immunofluorescence microscopy 3 days after plating (**G**,**H**,**I**). Double labeling of nestin (red) and βIII-Tub (green). Phase contrast and immunofluorescence images of cultures derived from neurospheres harvested at day 9 (**D**,**G**), day 16 (**E**,**H**) and day 23 (**F**,**I**). Scale bars: 100 μm.

Throughout differentiation, the aggregate size increased, reaching average diameters of 150 ± 40, 309 ± 94 and 458 ± 44 μm after 1, 2 and 3 weeks of RA treatment, respectively (Figure 5A, B, C, Table 2). The aggregate shape became uniform, forming compact and spherical structures (Figure 5B, C). Immunofluorescence microscopy of aggregate cryosections showed that these were neurospheres, composed of precursors (nestin-positive) and differentiated neurons (βIII-Tub-positive), the latest distributed preferentially at the surface (Figure 5C1).

**Table 2:** Characterization of NT2 neurospheres cultured in a fully controlled bioreactor.

**Neurospheres**			
Time of harvesting (day)	9	16	23
Duration of retinoic acid treatment (week)	1	2	3
Neurosphere size (μm)	150 ± 40	309 ± 94	458 ± 44
Differentiation efficiency	0.13 ± 0.06	17.2 ± 2.2	37.4 ± 0.9

Neurosphere size and neuronal differentiation efficiency are expressed as mean ± SEM from n = 2 independent bioreactor experiments.

After 9, 16 and 23 days of bioreactor culture (1, 2 and 3 weeks of neuronal differentiation, respectively), neurospheres were harvested and cultured for 7 days, on PDL-MG coated flasks, in MI medium, to allow cell migration and inhibit cell proliferation. One day post-seeding, the presence of neurites surrounding the neurospheres was more pronounced on cultures harvested at day 23 (Figure 5F), while on neurospheres harvested earlier, cells with flattened morphology predominated (Figure 5D). Three days post-seeding, the cell culture composition was analyzed by immunofluorescence microscopy (Figure 5G, H, I). Cultures derived from neurospheres harvested at day 23 were richer in neurons (βIII-Tub-positive staining) and presented more developed neuritic networks than the neurospheres harvested at day 16 (Figure 5H, I). A reduced number of βIII-Tub-positive cells was detected in cultures derived from neurospheres collected at day 9, in which nestin-positive cells predominated (Figure 5G). The estimated neuronal differentiation efficiency was 0.13 ± 0.06% and 17.2 ± 2.2% for cultures derived from neurospheres harvested at day 9 and 16 (Table 2). The results obtained until day 16 were similar to the ones described for the spinner culture [9], both in culture profile and differentiation efficiency, proving that the integrated culture strategy was successfully implemented in the bioreactor. Moreover, by extending the RA treatments for an additional week, a significant increase in the yield of neuronal differentiated cells was obtained (neuronal differentiation efficiency of 37.4 ± 0.9%, Table 2).

## Discussion

To fully fulfill the expectations raised by cell therapy it is urgent to develop robust and totally controlled culture systems, specially designed for the production of high numbers of differentiated and well characterized cells, expanded as fast and pure as possible. In the present study, we successfully developed a bioprocess for the rapid production of human neurons using fully controlled stirred tank bioreactors (125 mL). This was accomplished by integrating human NT2 cell expansion and differentiation in a two-step bioprocess.

In this particular study, an ideal expansion strategy should assure the fast production of high numbers of stem cells without compromising their potential. We demonstrated that, along expansion as 3-D aggregates, NT2 cells maintained their pluripotent and undifferentiated phenotype as well as the ability to differentiate into neurons. Different bioreaction parameters, including cell inoculum concentration and culture operation mode were studied. The results indicate 4 × 10^5 ^cell/mL as the most adequate inoculum strategy to be integrated with the differentiation step, as it allowed higher cell densities in less culture time contributing to a fast overall process. However, the feasibility of starting the cultures with inoculum concentrations as lower as 1 × 10^5 ^cell/mL looks promising for specific clinical applications in which the starting material is a limiting factor. Although lower inoculation concentrations have been used to expand undifferentiated murine embryonic stem cells as aggregates [6,24], NT2 cell proliferation could not be achieved when 4× 10^4 ^cell/mL were used. This difference in cell behavior may reflect the distinct cell origins, as NT2 are pluripotent human embryonal carcinoma stem cells, derived from teratocarcinomas [23], that closely resemble the human embryonic stem cells derived from the blastocyst inner cell mass [25].

By using a fed-batch strategy, where low levels of glucose were maintained in culture, it was possible to enhanced glucose metabolism efficiency with a concomitant improvement of the FI in cell concentration and increase of culture lifespan. This strategy may have minimized the toxicity effect associated with lactate accumulation, as reported previously for several animal cell cultures [21,26]. Nevertheless, the accumulation of other toxic metabolites, including ammonia, resulted in an increase in cell death. The possible depletion of nutrients (others than glucose and glutamine) as well as the exhaustion of essential small molecules, namely growth factors, not replenished in the glucose fed-batch strategy, may have contributed to arrest cell growth. The media exchange mode overcame these drawbacks, being the most efficient strategy to enhance undifferentiated stem cell cultivation, as shown by the higher cell densities and higher culture viability obtained throughout the cultivation time. Therefore this strategy was chosen for implementation in the controlled bioreactor in which stem cell expansion was successfully reproduced, confirming the robustness of the process. Media exchange and perfusion strategies have been used previously for adult stem cell cultivation [3,9] and human embryoid bodies [13]. In order to achieve higher expansion ratios, as those obtained for the expansion process as aggregates of murine embryonic stem cell [6,24] and human neuronal precursor cell [17], serial passage with addition of fresh media can be further included.

By incorporating both expansion and differentiation steps in an integrated bioprocess, this strategy also assures the feasibility of expanding human differentiated neurons derived from a continuous source of pluripotent stem cells. The system described herein allows for obtaining well differentiated neurons after 2 weeks of differentiation, as well as higher yields of neurons for a later culture time. Importantly, when compared to well established static differentiation protocols, this methodology drastically enhanced the neuronal differentiation efficiency of NT2 cells and reduced the time needed for differentiation process; for a differentiation time of 23 days in the bioreactor culture a 10-fold improvement in yield was observed over the static culture protocols lasting 35 days [23].

In this work, the expansion and differentiation of NT2 cells was successful validated in computer-controlled bioreactors. In future, further optimizations can be attempted aiming to determine the optimal conditions (pH, pO_2 _and temperature) to grow and differentiate NT2 cells. So far, some studies have demonstrated that low pO_2 _decreases the rate of stem cell differentiation and enhances stem cell proliferation [27]. Nieruebuegge *et al*. also reported a significant increase in final cell number as well as an improvement of cardiac-enriched genes in hEBs cultures under hypoxic conditions (pO_2 _= 4%) [7]. A recent study reports that rat mesenchymal stem cell differentiation is enhanced at lower temperatures (32°C) than in 37°C conditions [28].

## Conclusion

In this work, a scalable and efficient two-step bioprocess for the generation of human NT2-derived neurons was developed in a fully controlled bioreactor, allowing continuous monitoring, non-invasive sampling and characterization. By integrating a fast expansion step with an efficient differentiation process, this strategy significantly reduced the time and improved the yields of the neuronal differentiation, when compared to the standard static differentiation protocols.

The controlled bioprocess developed herein can be adaptable to other cell types, including hESCs and iPS, representing a strong and promising starting point for the development of novel technologies for the production of differentiated derivatives from pluripotent cells.

## Methods

### Cell culture

NTERA-2/cl.D1 cells (NT2) were obtained from the CNDR, University of Pennsylvania School of Medicine. Undifferentiated NT2 cells were routinely cultivated in standard tissue culture flasks (Nunc) and maintained in OptiMEM medium (Invitrogen) supplemented with 5% (v/v) of fetal bovine serum (FBS, Hyclone) and 100 U/mL of penicillin- streptomycin (P/S, Invitrogen), according to method described at Brito *et al*.[29].

### Stirred suspension culture

#### Undifferentiated NT2 cell expansion in spinner vessels

Undifferentiated NT2 cells (passage 60-62) were cultured as 3D-aggregates in 125-mL spinner vessels (Wheaton) equipped with a ball impeller and maintained at 37°C and 5% CO_2 _for up to 7 days. The agitation rate was increased during cultivation in order to avoid aggregate clumping and to control aggregate size (day 0 to 2 - 60 rpm, day 2 to 3 - 70 rpm, day 3 to 4 - 80 rpm, day 4 upwards - 90 rpm). Two independent experiments were performed for each expansion strategy.

##### Inoculum Concentration Experiments

Cells were cultured in a batch operation mode in Dulbecco's Modified Eagle's Medium- High Glucose (DMEM-HG, 25 mM glucose) (Invitrogen) supplemented with 10% (v/v) FBS and 100 U/mL of P/S (complete DMEM-HG). The cell inoculum concentrations evaluated were: 0.4 × 10^5^, 1 × 10^5 ^and 4 × 10^5 ^cell/mL; for an easier reading the nomenclature used was SP-0.4B, SP-1B and SP-4B, respectively. In SP-0.4B and SP-1B, cells were cultured in 75 mL of medium at 50 rpm during the first 4-8 h, to promote cell aggregation.

##### Culture Operation Mode Experiments

Glucose fed-batch and medium exchange culture operation modes were performed using an inoculum cell density of 4 × 10^5 ^cell/mL; the nomenclature used for these experiments were SP-4FB (SP- spinner, FB- fed-batch) and SP-4ME (SP- spinner, ME- media exchange), respectively. In SP-4FB, the culture medium was DMEM-Base (Sigma) supplemented with 10% (v/v) FBS, 4 mM of glutamine (Invitrogen), 100 U/mL P/S and 1.4 mM of glucose (Merck). During culture time, glucose concentration was monitored twice a day and maintained at lower levels (<1.4 mM); refeeds were performed accordingly to the consumption rates (calculated from 2 consecutive samples). SP-4ME was cultured in similar conditions to those described for SP-4B, except that medium was partially exchanged daily from the day 3 onwards as follows: fifty percent of culture media was collected in sterile conditions and centrifuged at 200 × *g *for 5 min; the supernatant was discarded and the recovered cell aggregates gently resuspended in an equivalent volume of pre-warmed complete DMEM-HG.

For all spinner cultures, sampling (2.5 mL) was performed 4 h after inoculation and daily from then on. Cell aggregates were monitored under an inverted microscope (Leica DM IRB). Cell concentration, metabolite concentration and lactate dehydrogenase activity were analyzed as described below.

#### NT2 culture in a fully controlled bioreactor

To ensure fully controlled cell culture environment, a stirred tank bioreactor [30] equipped with ball impeller and pH and dissolved oxygen (pO_2_) measuring probes (Mettler-Toledo) was used for the expansion and differentiation of NT2 cells. The pH was kept at 7.2 by injection of CO_2 _and addition of base (NaOH, 0.2 M). The pO_2 _was maintained at 25% via surface aeration. The temperature was kept at 37°C by water recirculation in the vessel jacket controlled by a thermocirculator module. Data acquisition and process control were performed using MFCS/Win Supervisory Control and Data Acquisition (SCADA) software (Sartorius-Stedim, Germany).

##### NT2 cell expansion

The SP-4ME experiment was reproduced in the bioreactor system, using undifferentiated NT2 cells with 60-62 passages in static conditions. Moreover, cells used for the inoculum (day 0) and at days 3 and 6 of cultivation in the bioreactor, were characterized using immunofluorescence tools and the neuronal differentiation potential evaluated (see below).

##### NT2 neuronal differentiation

Undifferentiated NT2 cells with up to 62 passages in static conditions were expanded in the bioreactor, in complete DMEM-HG, using an inoculum concentration of 4 × 10^5 ^cell/mL. Differentiation was initiated in the middle of the exponential phase (day 3), following the differentiation protocol developed by Serra et al [9]. Briefly, neuronal differentiation was induced by addition of retinoic acid (RA, Sigma) to the culture media, at a final concentration of 10 μM. A 50% media exchange was performed 3 times a week on a regular basis for up to 24 days. Two bioreactor independent experiments were performed.

Samples were collected from the bioreactor at 3 time points: day 9, 16 and 23 (corresponding to 1, 2 and 3 weeks of differentiation process). Cell concentration and neurosphere size were determined and culture was characterized using immunofluorescence microscopy. Neurospheres harvested at the referred time points were transferred to coverslips or culture flasks (5 × 10^4 ^cell/cm^2^) coated with poly-D-lysine (PDL, Sigma) and Matrigel (MG, Becton-Dickinson) and cultured for up to 7 days in mitosis inhibitor (MI) medium: DMEM-HG supplemented with 5% FBS, 100 U/mL of P/S, 1 μM cytosine arabinosine (Sigma), 10 μM fluorodeoxyuridine (Sigma) and 10 μM uridine (Sigma). Neurons were selectively trypsinized [22,23] using a 0.015% Trypsin-EDTA solution (prepared from Trypsin-EDTA 1X, liquid 0.05% Trypsin, Invitrogen), counted and transferred to coverslips coated with PDL and MG for characterization by immunocytochemistry. Neuronal differentiation efficiency was defined as the ratio between the number of neurons obtained after 7 days of culture in MI medium and the total amount of cells harvested at the 3 different harvesting times.

### Analytical methods

#### Cell concentration determination

Cell aggregates were dissociated by a 2 min incubation with Trypsin-EDTA (0.05%) at 37°C followed by cell resuspension in complete DMEM-HG. Cell density was assessed using a Fuchs-Rosenthal haemocytometer (Brand, Wertheim, Germany) and cell viability estimated by the standard trypan blue exclusion test.

#### Aggregate diameter

Aggregate size in each culture sample was determined using a micrometer coupled to an inverted microscope (Leica, DM IRB). Two perpendicular diameters of a minimum of 15 aggregates were measured and the average diameter was calculated. Aggregates less than 20 μm in diameter (generally cell doublets or triplets) were not considered for calculations as they represent a small percentage of the total cell number in culture.

#### Lactate dehydrogenase activity

Lactate dehydrogenase (LDH) activity from the culture supernatant was determined as an indirect way of assessing cell death. LDH activity was determined by following spectrophotometrically (at 340 nm) the rate of oxidation of NADH to NAD^+ ^coupled with the reduction of pyruvate to lactate. The specific rate of LDH release (q_LDH_, U.day^-1^.cell^-1^) was calculated for every time interval using the following equation: q_LDH _= ΔLDH/(Δt ΔX_V_), where ΔLDH (U) is the change in LDH activity over the time period Δt (day) and ΔXv (cell) is the average of total cells during the same time period. The cumulative value q_LDHcum _was estimated by q_LDHcum i+1 _= q_LDH i _+ q_LDH i+1_. The fold increase of the specific LDH release rates achieved throughout 6 days of cultivation were determined by calculating the ratio between the values of q_LDHcum _obtained at day 6 and day 0. These values indirectly represent the fold increase in cell lysis obtained within 6 days of culture.

#### Metabolite analysis

Glucose (GLC), lactate (LAC) and glutamine (GLN) concentrations in the culture medium were analyzed using an YSI 7100MBS (YSI Incorporated, USA). Ammonia was quantified enzymatically using a commercially available UV test (Roche, Germany).

The specific metabolic rates (*q*_Met_., mol.day^-1^.cell^-1^) were calculated using the equation: q_Met_. = Δ_Met_/(Δt ΔX_v_), where Δ_Met _(mol) is the variation in metabolite concentration during the time period Δt (day) and ΔX_v _(cell) the average of adherent cells during the same time period.

#### Apparent growth rate and fold increase in cell expansion

Apparent growth rates and fold increase parameters were calculated for all expansion cultures. Apparent growth rates (μ, day^-1^) were calculated using a first order kinetic model for cell expansion: dX/dt = μX, where t (day) is the culture time and × (cell) is the value of viable cells for a specific t. The μ values were estimated applying the model to the slope of the curves during the exponential phase. The fold increase in cell expansion (FI) was defined as the ratio X_MAX_/X_0_, where X_MAX _is the peak cell density (cell/mL) and X_0 _is the inoculation cell density (cell/mL).

#### Differentiation potential

To assess the neuronal differentiation potential along the expansion assays, 2.3 × 10^6 ^cells were collected from the suspension cultures and plated in a T75 flask (Nunc) (Figure 1). NT2 cells were differentiated into post-mitotic neurons according to Pleasure et al [23]. Briefly, cells were cultured for 5 weeks in complete DMEM-HG supplemented with 10 μM RA. Cells were splitted at 1:4.5 ratio and cultured in MI medium for 12 days. After this period, neurons were selectively trypsinized, as described above, counted and transferred to coverslips coated with PDL and MG for characterization by immunocytochemistry. Neuronal differentiation efficiency was defined as the ratio between the number of neurons obtained after culture in MI medium and the total amount of cells harvested after RA treatments.

#### Immunofluorescence microscopy

In expansion cultures, cell aggregates were collected at day 3 and 6, dissociated using Trypsin-EDTA (0.05%) at 37°C followed by cell resuspension in complete DMEM-HG, and transferred to glass coverslips. Three days after plating, cultures were characterized. In differentiation assays neurospheres were harvested from the bioreactor cultures at day 9, 16 and 23, and processed for cryosection or transferred to coverslips coated with PDL and MG (see Figure 1).

Cells in coverslips were washed in PBS with 0.5 mM MgCl_2 _and fixed in 4% (w/v) paraformaldehyde solution in PBS with 4% (w/v) sucrose, for 20 min. For cryosection, neurospheres were washed in PBS, transferred to a tissue protecting compound (Tissue Teck, OCT™ Compound) and frozen at -80°C. Ten μm sections, obtained using a cryostat (Leica), were rehydrated with PBS and fixed in methanol, at -20°C, for 10 min. After fixation, the same procedure was followed for cryosections and coverslips.

For staining intracellular epitopes, cells were permeabilized with 0.1% (w/v) Triton X-100 (TX-100) in PBS, for 15 min. After 1 h in blocking solution (0.2% (w/v) fish skin gelatin in PBS), cells were incubated with primary antibody for 2 h. The coverslips were washed 3 times with PBS and overlaid with secondary antibody for 1 h. Primary and secondary antibodies were diluted in 0.125% (w/v) fish skin gelatin in PBS with 0.1% (w/v) TX-100. Samples were mounted in ProLong mounting medium (Molecular Probes), supplemented with DAPI for nucleus staining. For surface epitopes staining, cells were not permeabilized with TX-100. Samples were visualized using a fluorescence microscope (Leica DMRB).

Primary antibodies used were: mouse anti-tumor related antigen-1-60 (Tra-1-60) (Santa Cruz Biotechnology), mouse anti-stage specific embryonic antigen-4 (SSEA-4) (Santa Cruz Biotechnology), mouse anti-Oct-4 (Santa Cruz Biotechnology), mouse anti-nestin (Chemicon), mouse anti-type III β-tubulin (βIII-Tub) (Chemicon), mouse anti-microtubule associated protein 2A and 2B (MAP2) (Chemicon). The secondary antibodies were goat anti-mouse IgM-AlexaFluor488, goat anti-mouse IgG-AlexaFluor 594, goat anti-mouse IgG-AlexaFluor 488 and rabbit anti-mouse IgG-AlexaFluor 594 (Invitrogen).

#### Flow cytometry

Cells used for the inoculum (day 0) and from day 3 of the bioreactor expansion culture were dissociated into single cells and analyzed by flow cytometry (Figure 1). Samples were fixed in CytofixCytoperm reagent (BD Pharmigen) for 10 min, blocked with 1% BSA in PBS at 4°C for 30 min and, in the case of intracellular antigens, permeabilized with 1% TX-100 for 10 min. Primary antibodies were mouse anti-Tra-1-60 and anti-Oct-4. Secondary antibodies were anti-mouse IgM-AlexaFluor488 and anti-mouse IgG-AlexaFluor488. Ten thousand events were registered per sample with a CyFlow^® ^space (Partec) instrument, using the appropriate scatter gates to avoid cellular debris and aggregates. A cell was considered to be positively stained if the measured fluorescence intensity exceeded the signal obtained by cells incubated with an isotype control antibody (Santa Cruz Biotechnology).

### Statistical analysis

For each spinner and bioreactor assays, two independent experiments were performed. The results were expressed as the mean ± standard deviation. The statistical test used, One-way ANOVA, was performed in SPSS 13.0 for Windows for a level of confidence of 95% (a = 0.05) followed by the Scheffé multiple comparison test.

## List of abbreviations

BSA: bovine serum albumin; DAPI: 4',6-diamidino-2-phenylindole; DMEM-HG: Dulbecco's modified Eagle's medium-high glucose; FBS: foetal bovine serum; FI: fold increase; hESC: human embryonic stem cells; iPS cells: induced pluripotent stem cells; LAC: lactate; LDH: lactate dehydrogenase; MAP2: microtubule-associated protein 2; MG: Matrigel; MI: mitosis inhibitors; NT2: NTera2/cl.D1; P/S: penicillin-streptomycin; PDL: poly-D-lysine; pO_2_: dissolved oxygen; q_GLC_: specific rate of glucose consumption; q_LAC_: specific rate of lactate production; q_LDHcum_: cumulative value of specific LDH release rate; RA: retinoic acid; SSEA-4: stage specific embryonic antigen-4; Tra-1-60: tumor related antigen-1-60; TX-100: triton X-100; βIII-tub: type III β-tubulin; μ: apparent growth rate.

## Authors' contributions

MS participated in the spinner and bioreactor experiments and in the collection of Flow Cytometry data; carried out the growth kinetics and metabolic profile analyses and the cryosection immunofluorescence microscopy; contributed to the conception and design of the study and drafted the manuscript. CB participated in the spinner and bioreactor experiments and immunological assays; carried out the assessment of the neuronal differentiation potential; contributed to the conception, design and coordination of the study and helped to draft the manuscript. EC was involved in spinner experiments and immunofluorescence microscopy analysis. MFQS participated in bioreactor experiments. PA participated in the conception, design and coordination of the study and gave final approval of the version to be published. All authors read and approved the final manuscript.
